# 

**Published:** 1903-01-15

**Authors:** 


### CONTENTS.

Dental Embryology, - *By G. S. Blanchard, D.D.S.* 1

Modern Use of Soft Foil, *By G. S. Junkerman, M.D., D.D.S.* 10

The Radical Preparation of Cavities, *By Harry M. Semans, D.D.S.* 17

President's Address, - - *By Otto Arnold, D.D.S.* 29

Teeth in Relation to Degeneracy, *By H. C. Rutter, M. D.* 36

EDITORIAL, - - - - - - - - - - 46

INDENTS, - - - - - - - - - - 50

